# 

**Published:** 1893-04

**Authors:** 


### LITERARY.

A HAND-BOOK OF INVALID COOKING.—"For the use of Nurses and others who Care for the Sick." By Mary A. Boland. Instruction of Cooking, etc., etc. 4to, 323 pp. The Century Co., Publishers.

This book is just what it purports to be: a Hand-Book for Nurses, and no nurse should be without it.

Part 1st consists in a series of Explanatory Lessons on the properties of various foods adapted to the sick, and with such other needful information as tends to fit one for a practical nurse.

Part 2d contains a well arranged body of recipes, with brief explanations of their elements and adaptability in various disorders and periods of life. An excellent and timely work, which every family should have.

PERIODICALS DESIGNED FOR YOUTH.—No country is in advance of ours in furnishing suitable periodical reading for the young. Here we have Baby Land, Our Little Men and Women, Youth's Companion, and a host of others, among the more interesting being The Young Sportsman, a monthly, published at Albion, N. Y., replete with stories of adventure and tales of wild animals.

COLLEGE EXPENSES OF STUDENTS.—Frank Booles, Secretary of Harvard University, has made and published various tabulated statements of students' expenses, for the college year, the average being about $750, including board.

The pamphlet is useful as showing for what an inconsiderable sum the college course can be taken.

THE WORLD ALMANAC, which we offer as a premium to our $1.00 subscribers is indeed a useful encyclopædia of current events of the past year everywhere in the wide world, and very much useful information respecting things which everybody is presumed to know all about. See our premium list.

